# Sox9 Potentiates BMP2-Induced Chondrogenic Differentiation and Inhibits BMP2-Induced Osteogenic Differentiation

**DOI:** 10.1371/journal.pone.0089025

**Published:** 2014-02-13

**Authors:** Junyi Liao, Ning Hu, Nian Zhou, Liangbo Lin, Chen Zhao, Shixiong Yi, Tingxu Fan, Wei Bao, Xi Liang, Hong Chen, Wei Xu, Cheng Chen, Qiang Cheng, Yongming Zeng, Weike Si, Zhong Yang, Wei Huang

**Affiliations:** 1 Department of Orthopaedic Surgery, The First Affiliated Hospital of Chongqing Medical University, Chongqing, China; 2 Department of Radiology, The First Affiliated Hospital of Chongqing Medical University, Chongqing, China; 3 Department of Clinical Hematology, Third Military Medical University, Chongqing, China; Georgia Regents University, United States of America

## Abstract

Bone morphogenetic protein 2 (BMP2) is one of the key chondrogenic growth factors involved in the cartilage regeneration. However, it also exhibits osteogenic abilities and triggers endochondral ossification. Effective chondrogenesis and inhibition of BMP2-induced osteogenesis and endochondral ossification can be achieved by directing the mesenchymal stem cells (MSCs) towards chondrocyte lineage with chodrogenic factors, such as Sox9. Here we investigated the effects of Sox9 on BMP2-induced chondrogenic and osteogenic differentiation of MSCs. We found exogenous overexpression of Sox9 enhanced the BMP2-induced chondrogenic differentiation of MSCs in vitro. Also, it inhibited early and late osteogenic differentiation of MSCs in vitro. Subcutaneous stem cell implantation demonstrated Sox9 potentiated BMP2-induced cartilage formation and inhibited endochondral ossification. Mouse limb cultures indicated that BMP2 and Sox9 acted synergistically to stimulate chondrocytes proliferation, and Sox9 inhibited BMP2-induced chondrocytes hypertrophy and ossification. This study strongly suggests that Sox9 potentiates BMP2-induced MSCs chondrogenic differentiation and cartilage formation, and inhibits BMP2-induced MSCs osteogenic differentiation and endochondral ossification. Thus, exogenous overexpression of Sox9 in BMP2-induced mesenchymal stem cells differentiation may be a new strategy for cartilage tissue engineering.

## Introduction

From degenerative disorders to traumatic injuries, cartilaginous pathologies present a very significant clinical challenge to the medical fraternity especially due to its lack of regenerative capabilities [1]. To overcome this drawback, many surgical interventions were applied. Of these methods, the 2 important surgical methods designed to promote cartilage repair were the bone marrow stimulation techniques and restoration techniques [2], [3], [4]. But, the bone marrow stimulation techniques such as microfracture and drilling produce fibrocartilage with insufficient long-term effects [5], [6]. Restoration techniques such as autologous chondrocyte implantation and osteochondral allograft were limited by insufficient cell supply, damage to the donor site, and immunological reactions [7], [8]. As a consequence, stem cell based and gene-enhanced tissue engineering cartilage is considered to be more promising in the treatment of cartilaginous pathologies [7], [9].

Mesenchymal stem cells (MSCs) can undergo self-replenishment and have the potential to differentiate into multiple lineages, including osteogenic, chondrogenic, and adipogenic lineages [9], [10], [11], [12]. Due to the abundant source, easy isolation, and stable expression of the exogenous genes, MSCs have been regarded as ideal seed cells for scientific research on cartilage tissue engineering [12]. Many studies have reported that different growth factors such as BMPs [13], FGFs [14], [15], IGF1 [16], TGF-β [17], [18] have been identified for their ability to direct MSCs towards the chondrocyte phenotypes [19]. However, the use of these growth factors is still disputable, because of their limited ability towards the synthesis of specific cartilage matrix components. Hence, optimizing a chondrogenic growth factor and amplifying its specific chondrogenic ability is one of the most crucial and key step in the process of cartilage tissue engineering.

Bone morphogenetic protein 2 (BMP2), belonging to the transforming growth factor beta (TGF-β) super-family, is known to induce human bone mesenchymal stem cells (hBMSCs) [14], adipose-derived stem cells (ADSCs) [16], mouse embryonic fibroblasts (MEFs) [20] chondrogenic differentiation, and it promotes MSCs condensation, chondrogenic differentiation, chondrocytes proliferation and hypertrophic differentiation [13]. BMP2 has a greater potential to induce chondrogenic differentiation of MSCs compared with other growth factors such as TGF-β, IGF1 etc [21], [22]. However, BMP2 is also known to induce MSCs osteogenic differentiation and stimulates endochondral ossification [23], [24]. Thus, potentiating BMP2-induced MSCs chondrogenic differentiation and inhibiting BMP2-induced MSCs osteogenic differentiation may play a vital role in chondrogenesis and cartilage formation.

In the present study, we made an attempt to investigate whether overexpression of Sry-related transcription factor Sox9 could potentiate BMP2 induced chondrogenic differentiation of MSCs, and inhibit osteogenic differentiation of MSCs. Sox9 is a transcription factor belonging to the Sry-related high-mobility-group box (Sox) proteins family [25]. It is essential for chondrogenesis of MSCs [26], [27], [28], since it is considered as the key transcription factor for BMP2 induced chondrogenesis [20]. It is also reported that Sox9 inhibits the transactivation of Runt-related transcription factor 2 (Runx2) [29], which is a key transcription factor for osteogenesis and endochondral ossification [30], [31]. However, it is not clear whether Sox9 mediated inhibition of osteogenic differentiation signaling plays any role in the BMP2 induced differentiation of MSCs. Nevertheless, we found that BMP2 induced Sox9 expression was transient and relatively at a lower level during the early stages of MSCs differentiation. Exogenous overexpression of Sox9 enhanced the BMP2 induced chondrogenic differentiation and markers expression, and inhibited BMP2 induced osteogenic differentiation and markers expression. In stem cell implantation studies, Sox9 was shown to potentiate BMP2 induced cartilage formation, and inhibit endochondral ossification during ectopic bone/cartilage formation. Through perinatal limb explant culture, we demonstrated that Sox9 and BMP2 synergistically promoted chondrocytes condensation and proliferation. However, Sox9 inhibited BMP2 induced chondrocytes hypertrophy, and ossification. Our findings strongly suggest that overexpression of Sox9 in BMP2 induced MSCs differentiation may become a new strategy for cartilage tissue engineering.

## Materials and Methods

### Ethics Statement

The experimental protocols were approved by the Ethical Committee of The First Affiliated Hospital of Chongqing Medical University. All animal protocols were approved by Ethical Committee of The First Affiliated Hospital of Chongqing Medical University. All surgery was performed under sodium pentobarbital anesthesia, and all efforts were made to minimize suffering.

### Cell Culture and Chemicals

The HEK 293 and C3H10T1/2 cell lines were obtained from ATCC (Manassas, VA). Cell lines were preserved in complete Dulbecco’s modified Eagle’s medium (DMEM, Hyclone, China), supplemented with 10% fetal bovine serum (FBS, Gibco, Australia), 100 U/ml penicillin, and 100 mg/ml streptomycin, maintained at 37°C in a humidified 5% carbon dioxide (CO_2_) atmosphere. Unless indicated otherwise, all chemicals were purchased from Sigma-Aldrich or Corning.

### Recombinant Adenoviruses Expressing GFP, BMP2 and Sox9

Recombinant adenoviruses were generated using AdEasy technology as described previously [32], [33]. The coding regions of GFP, BMP2, and Sox9 were amplified with PCR, and cloned into adenoviral shuttle vectors. Then the vectors were used to generate recombinant adenoviruses in HEK 293 cells. The resulting adenoviruses were designated as AdGFP, AdBMP2, and AdSox9. AdGFP was used as a vector control.

### Chondrogenic Differentiation of MSCs in Micromass Culture

The C3H10T1/2 cells transduced with AdGFP, AdBMP2 and/or AdSox9 were cultured in 100 mm dishes. At 80% confluence, cells were harvested and resuspended in the high-density (10^5^ cells in a 10 µl drop of media) culture medium. Then the medium (50 µl in each well) was added at the center of each well in the 12-well plates. The plates were then carefully transferred to CO_2_ incubators and incubated for 2 hours. About 2 to 3 ml of the medium was added to each well. Fresh medium was added to the wells every 4 to 5 days. Chondrogenic assays were carried out at desired time points.

### Osteogenenic Differentiation of MSCs in Monolayer Culture

The C3H10T1/2 cells were seeded in 6-well or 24-well plates, at 40 to 50% confluence. Cells were infected with AdBMP2 and/or AdSox9, and AdGFP was used as control. Fresh medium was added to the wells every 4 to 5 days. Cells were harvested at desired time points for subsequent analysis.

### Alcian Blue Staining for Micromass Pellet

Micromass cell pellet was washed with phosphate buffered saline (PBS), treated with 4% paraformaldehyde for 30 minutes, and again washed with PBS. The pellet was stained with 0.5% Alcian blue in 0.1 M HCl (pH 1.0) for 12 hours, and washed with distilled water. The pellet was then photographed with Nikon microscope. The Alcian blue-stained cultures were extracted at room temperature using the 6 M guanidine hydrochloride. Optical density (OD) of the extracted dye was measured at 630 nm in a microplate reader (Bio-Rad, America). All the experiments were performed in triplicate. All the results are represented as mean±standard deviation (SD).

### RNA Isolation and Semi-quantitative RT-PCR

The total RNA of the cells was isolated using TRIZOL reagent (Invitrogen, America) according to the manufacturer’s instructions. Reverse transcription reactions were performed using PrimeScript RT reagent kit (Takara, Dalian, China) according to the manufacturer’s instructions. The first strand cDNA products were further diluted ten times and used as PCR templates. Semi-quantitative PCR was performed using TaKaRa Ex Taq (Takara, Dalian, China), a touchdown cycling program, which is as follows: 95°C for 3 minutes for 1 cycle; 95°C for 30 seconds, 58°C for 30 seconds, and 72°C for 13 cycles and decreasing 0.5°C per cycle; and then at 95°C for 30 seconds, 58°C for 30 seconds, and 72°C for 30 seconds for 20–25 cycles; 72°C for 7 minutes; and finally hold at 4°C. PCR products were resolved on 1.5% agarose gels. All samples values were normalized to GAPDH expression. The primer sequences used for this analysis are listed in Table 1.

**Table 1:** Primer Oligonucleotide Sequences Used for PCR.

Gene	Forward Primer (5′-3′)	Reverse Primer (5′-3′)	Product Size
Aggrecan	TGGCTTCTGGAGACAGGACT	TTCTGCTGTCTGGGTCTCCT	188 bp
BMP2	ACCAGACTATTGGACACCAG	AATCCTCACATGTCTCTTGG	174 bp
Col2a1	CAACACAATCCATTGCGAAC	TCTGCCCAGTTCAGGTCTCT	159 bp
GAPDH	CTACACTGAGGACCAGGTTGTCT	TTGTCATACCAGGAAATGAGCTT	123 bp
Osteocalcin	CCTTCATGTCCAAGCAGGA	GGCGGTCTTCAAGCCATAC	161 bp
Osteopontin	CCTCCCGGTGAAAGTGAC	CTGTGGCGCAAGGAGATT	124 bp
Runx2	CCGGTCTCCTTCCAGGAT	GGGAACTGCTGTGGCTTC	122 bp
Sox9	AGCTCACCAGACCCTGAGAA	TCCCAGCAATCGTTACCTTC	200 bp

### Real-time PCR

The total RNA of the cells was isolated using TRIZOL reagent (Invitrogen, America) according to the manufacturer’s instructions. Reverse transcription reactions were performed using iScript cDNA synthesis kit (Bio-Rad, America) according to manufacturer’s instructions. Real-time PCR was performed using SsoAdvanced SYBR Green Supermix (Bio-Rad, America). The conditions maintained for real-time PCR are as follows: 95°C for 3 minutes for 1 cycle; 95°C for 10 seconds, 58°C for 5 seconds for 40 cycles. Dissociation stage was applied at the end of the amplification procedure. The dissolve curve did not determine any nonspecific amplification. All sample values were normalized to GAPDH expression by using the 2^−ΔΔCt^ method. The primer sequences used for this analysis are listed in Table 1.

### Immunocytochemical Staining

The C3H10T1/2 cells were infected with AdGFP, AdBMP2, and/or AdSox9. At desired time points, the cells were treated with 4% paraformaldehyde for 30 minutes at room temperature. After washing with PBS, the fixed cells were treated with 1% NP-40 and 10% goat serum, to render the cells more permeable. Following this step, the cells were incubated with primary antibodies against osteopontin (OPN) (Santa Cruz Biotechnology, America) at 4°C overnight. These cells were again washed with PBS, and were incubated with biotin containing secondary antibodies for 30 minutes, followed by incubation with streptavidin labeled horseradish peroxidase for 15 minutes at room temperature. The presence of the expected protein was visualized by DAB staining. Stains with IgG were used as negative controls. The results were repeated in at least 3 independent experiments.

### Western Blot Analysis

The cell lysates were prepared using cell lysis buffer containing a protease inhibitor PMSF (Beyotime, Shanghai, China). About 60 µg of total protein for each sample was loaded onto 10% SDS-PAGE and transferred to PVDF membrane. The membrane was incubated overnight with antibodies against Sox9 (Santa Cruz Biotechnology, America), collagen type II alpha 1 (Col2a1) (Santa Cruz Biotechnology, America), OPN (Santa Cruz Biotechnology, America), osteocalcin (OC) (Santa Cruz Biotechnology, America) and β-actin (Bioworld Technology, American) at a dilution of 1∶500 or 1∶1000, respectively., Following this, the membrane was again incubated with a secondary antibody conjugated with horseradish peroxidase (Earthox, America). Immune-reactive signals were detected using ECL kit (Millipore, America).

### Alkaline Phosphatase (ALP) Assay

The ALP activities were assessed using the modified Great Escape SEAP chemiluminescence assay (BD Clontech) and/or histochemical staining, as described previously [34], [35]. For ALP histochemical staining, the cells were induced for osteogenic differentiation using AdGFP, AdBMP2 and/or Sox9, and DMSO (solvent control) as control. Infected cells were fixed with fixative solution (2 volumes of citrate working solution to 3 volumes of acetone) at room temperature for 30 seconds. After washing with distilled water, cells were stained subjected to histochemical staining with a mixture of 0.1 mg/mL of napthol AS-MX phosphate and 0.6 mg/mL of Fast Blue BB salt. Histochemical staining was recorded using bright light microscopy.

For the chemiluminescence assays, each assay condition was performed in triplicate, and the results were repeated in at least 3 independent experiments. The ALP activity was normalized by total cellular protein concentrations among the samples.

### Matrix Mineralization Assay (Alizarin Red S Staining)

The C3H10T1/2 cells were seeded in 24-well plates and infected with AdGFP, AdBMP2, and/or AdSox9. Infected cells were cultured in the presence of ascorbic acid (50 mg/ml) and β-glycerophosphate (10 mM). On day 14 after infection, mineralized matrix nodules were stained for calcium precipitation by means of alizarin red S staining, as described previously [34], [35], [36]. Briefly, cells were treated with 4% paraformaldehyde for 30 minutes. After washing with PBS, cells were incubated with 2% alizarin red S for 30 minutes, followed by extensive washing with distilled water. The staining of calcium mineral deposits was recorded using bright light microscopy.

### Subcutaneous Stem Cell Implantation (Ectopic Cartilage/Bone Formation Assay)

The C3H10T1/2 cells were infected with AdGFP, AdBMP2, and/or AdSox9. Twenty four hours after infection, cells were harvested and resuspended in PBS containing 300 U/ml penicillin, and 300 mg/ml streptomycin for subcutaneous injection (5×10^6^ per injection) into the flanks of athymic nude (nu/nu) mice (3 animals per group, 4- to 6-week old males, Experimental Animal Center, Chongqing Medical University, Chongqing, China). Animals were euthanized, and the implantation sites were retrieved for histological and other staining evaluations at 5 and 8 weeks, respectively.

### Mouse Fetal Limb Explant Culture

The forelimbs of mouse embryos (E18.5) were dissected under sterile conditions and incubated in DMEM (Hyclone, China) containing 0.5% FBS (Gibco, Australia), 50 mg/ml ascorbic acid, 1 mM β-glycerophosphate, 100 U/ml penicillin, and 100 µg/ml streptomycin at 37°C in humidified air with 5% CO_2_ for up to 14 days, as described previously [37], [38]. The limbs were infected by AdGFP, AdBMP2, and/or AdSox9 directly, 12 hours after dissection. About 50% of the medium was replaced every 2 to 3 days. Cultured tissues were observed at different time points under the microscope to confirm the survival of tissue cells and the expression of fluorescence markers.

### Histologic Evaluation: Hematoxylin and Eosin, Masson’s Trichrome, Alcian Blue and Safranin O-fast Green Staining

Retrieved and cultured tissues were treated with 10% formalin, decalcified and embedded in paraffin. Paraffin-embedded sections were deparaffinized and then rehydrated in a graduated fashion. The deparaffinized samples were subjected to antigen retrieval and fixation. The sections were stained with hematoxylin and eosin (H&E), Masson’s trichrome, Alcian blue and Safranin O-fast green. Histological evaluation was performed using a light microscope (Nikon).

### Statistical Analysis

All quantitative experiments were performed in triplicate and/or repeated 3 times. Data were expressed as mean ± standard deviation (SD). The one-way analysis of variance was used to analyze statistical significance. A value of *P*<0.05 was considered statistically significant.

## Results

### Gene Transduction of MSCs and Expression of Sox9 in Each Treatment Groups

**Figure 1:**
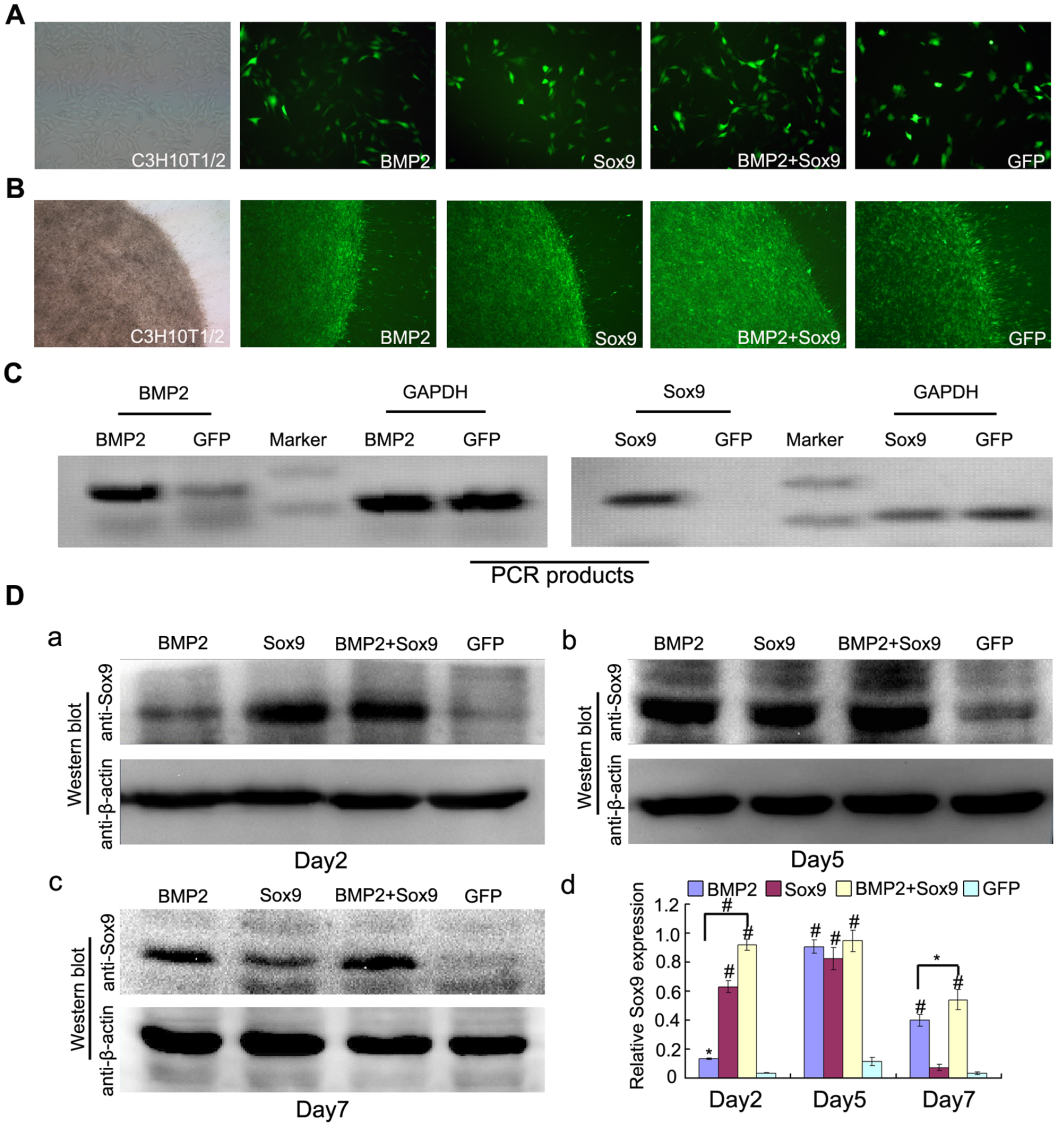
Gene transduction and Sox9 expression in each treatment groups. (A–B): Bright light and fluorescence microscope examination showed the transduction efficiency of recombinant adenoviruses in monolayer culture (24 hours after transduction, 100X) and micromass culture (3 days after transduction, 40X), respectively. (C): Recombinant adenoviruses mediated overexpression of BMP2 and Sox9 mRNA were evaluated by semi-quantitative RT-PCR analysis using GAPDH as a house keeping gene. (D): Sox9 expression were evaluated by western blot analysis in each treatment group at day 2, 5, and 7 after transduction (a to c) and relative Sox9 expression were analyzed by quantity one software using β-actin as controls (d), the results are expressed as mean±SD of triplicate experiments, *P<0.05, ^#^ *P<0.01*.

The C3H10T1/2 cells were infected by recombinant adenoviruses expression GFP, BMP2, and/or Sox9 successfully both in monolayer and micromass culture (Figures 1A, 1B). The BMP2 and Sox9 mRNA were upregulated at 36 hours after BMP2 and Sox9 transduction using semi-quantitative RT-PCR analysis (Figure 1C). Expression of Sox9 after infection was evaluated by western blot technique on day 2, day 5, and day 7 after incubation in a normal medium without any supplemented growth factors in micromass cultue (Figure 1D: a–c). Some studies have reported that Sox9 could be upgegulated by BMP2 [13], [20]. We found that Sox9 expression induced by BMP2 was time-dependent and showed a highest level on day 5. When combined with Sox9, it showed a significant earlier onset of overexpression of Sox9 (day 2) and maintained significant values at high level from day 2 to day 7 (Figure 1D: d). These results suggest an efficient and relatively sustained overexpression of Sox9 in BMP2 and Sox9 co-infected C3H10T1/2 cells.

### Sox9 Potentiates BMP2-induced Chondrogenic Differentiation of MSCs in Micromass Cultures in vitro

**Figure 2:**
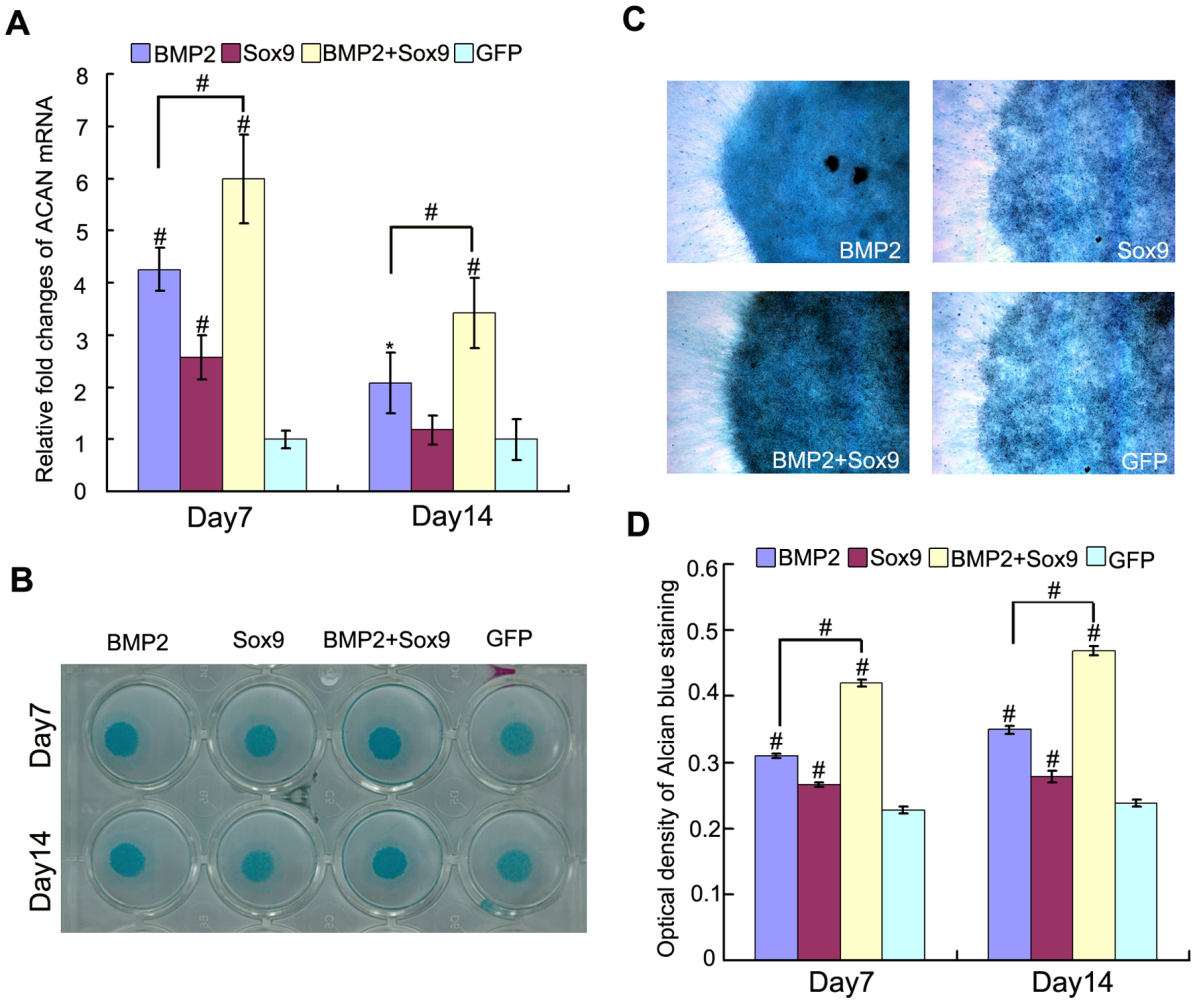
Sox9 potentiates BMP2-induced glycosaminoglycans synthesis in MSCs in micromass cultures. (A): Real-time PCR for the expression of chondrogenic differentiation marker gene ACAN were conducted on day 7 and day 14 after infection of AdGFP, AdBMP2, and/or AdSox9, using GAPDH as a house keeping gene. (B–C): Alcian blue staining for sulfated glycosaminoglycans in micromass cultures of C3H10T1/2 cells on day 7 and day 14 after transduction of indicated recombinant adenoviruses, gross observation (B) and microscope examination (C, 40X) are shown. (D): Alcian blue staining quantifying: cells were extracted with 6 M guanidine hydrochloride, Optical density of the extracted dye was measured at 630 nm. The results were expressed as mean±SD of triplicate experiments, *P<0.05, ^#^ *P<0.01*.

Since cell density is necessary for chondrogenic differentiation of MSCs, and micromass culture could provide cell to cell contact in 3-dimensions (3D), which is similar to MSCs condensation to induce chondrogenesis in vivo [39]. So, micromass cultures were used to evaluate the influence of exogenous overexpression of Sox9 in BMP2-induced chondrogenic differentiation of MSCs in vitro. Glycosaminoglycans and Collagen Type II alpha 1 (Col2a1) are the 2 main markers of chondrocyte and formed cartilage matrix. Therefore, we evaluated the 2 markers in micromass culture in vitro. As shown in Figure 2A, aggrecan (ACAN) mRNA was upregulated by BMP2 (*P*<0.05), and Sox9 enhanced this effect (*P*<0.01) on day 7 and day 14, respectively. Alcian blue staining was used to detect the sulfated glycosaminoglycans on day 7 and day 14. Gross observation and microscopic examination are shown in Figures 2B and 2C. Alcian blue staining quantification was also used to evaluate the chondrogenic differentiation of MSCs. The synergistic effect of Sox9 on BMP2-induced chondrogenic differentiation were observed on day 7 and day 14, respectively (*P*<0.01) (Figure 2D).

**Figure 3:**
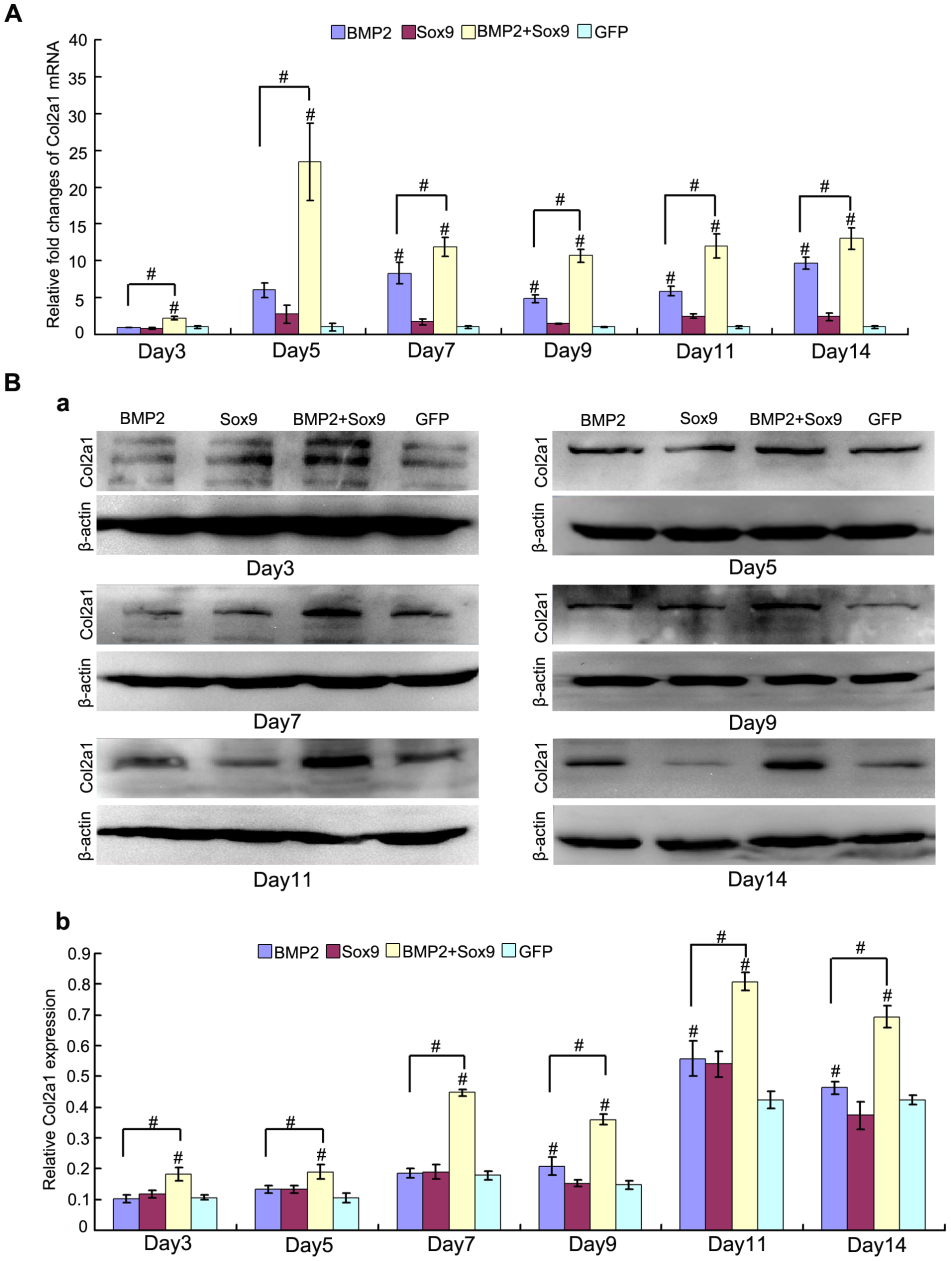
Sox9 potentiates BMP2-induced Col2a1 synthesis in MSCs in micromass cultures. (A): Real-time PCR for the expression of chondrogenic differentiation marker gene Col2a1 were conducted at continuously time points (from day 3 to day 14) after infection of AdGFP, AdBMP2, and/or AdSox9, using GAPDH as a house keeping gene. (B) Western blot for the expression of Col2a1 were conducted at continuously time points (from day 3 to day 14) after transduction of indicated recombinant adenoviruses (a), quantitatively, relative Sox9 expression were analyzed by quantity one software using β-actin as controls (b). The results are expressed as mean±SD of triplicate experiments, *P<0.05, ^#^ *P<0.01*.

Col2a1 is one of the most important molecular markers for chondrogenesis. We evaluated the expression of Col2a1 both on mRNA and protein level at continuous time points (Figure 2). While exogenous overexpression of Sox9 alone did not exert any significant effect on Col2a1 mRNA and protein expression, Sox9 was shown to exhibit a synergistic effect on BMP2-induced Col2a1 expression on both mRNA (Figure 3A) and protein level (Figure 3B). Interestingly, BMP2 failed to upregulate Col2a1 mRNA and protein expression at early stage (day 3, and day 5) of MSCs differentiation (*P*>0.05). However, when combined with Sox9, they showed significant earlier onset of expression of Col2a1 (day3), and they maintained their significant values at high level at all time points (from day 3 to day 14) both on mRNA (Figure 3a) and protein level (Figure 3Bb) (*P*<0.01).

These results show that exogenous overexpression of Sox9 potentiates BMP2-induced chondrogenic differentiation of MSCs in vitro persistently.

### Sox9 Inhibits BMP2 Induced Osteogenic Differentiation of MSCs in vitro

**Figure 4:**
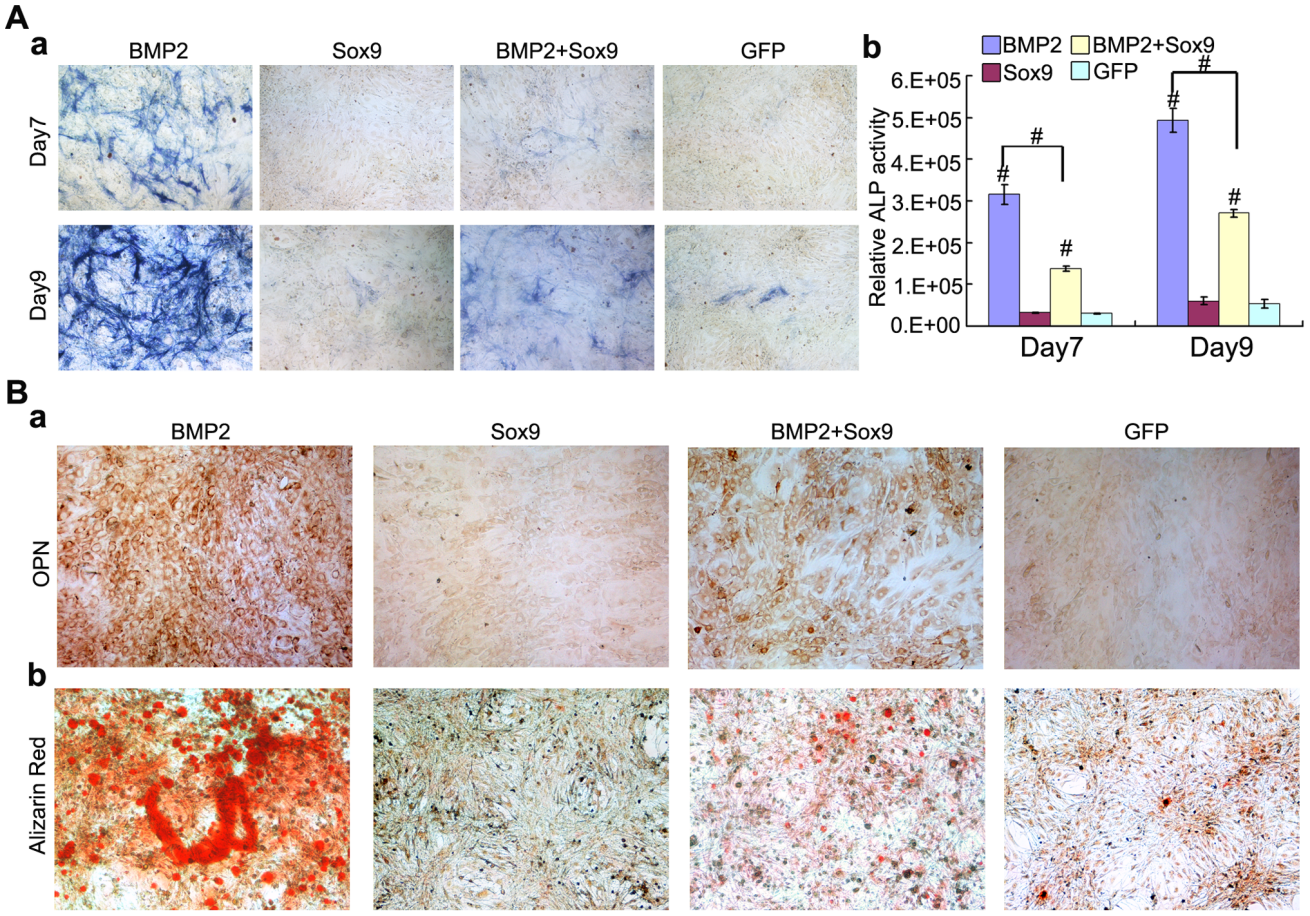
Sox9 inhibits BMP2-induced early and late osteogenic differentiation of MSCs in vitro. (A): C3H10T1/2 cells were infected with AdGFP, AdBMP2 and/or AdSox9. The ALP activities were measured on day 7 and day 9 using ALP histochemical staining (a), and chemiluminescent assays (b). (B): C3H10T1/2 cells were infected with indicated recombinant adenoviruses. On day 11 after infection, the expression of osteopontin (OPN) was assayed by immunocytochemical staining using anti-OPN antibody (a). For matrix mineralization, C3H10T1/2 cells were infected with indicated recombinant adenoviruses and cultured in mineralization medium. Alizarin Red staining were conducted on day 14 after infection (b). Each assay was done in triplicate. ALP assays results are expressed as mean±SD, *P<0.05, ^#^ *P<0.01*.

BMP2 also induces MSCs osteogenic differentiation and upregulates osteogenic markers at the late stage of chondrogenesis. We studied the effect of exogenous overexpression of Sox9 on BMP2-induced MSCs osteogenic differentiation. ALP activity is an early marker of osteogenic differentiation. ALP activity was measured on day 7 and day 9 after transduction. As expected, BMP2 induced a significant increase in ALP activity (*P*<0.01), especially on day 9 (Figure 4a). However, when co-infected with Sox9, ALP activity decreased dramatically both on day 7 and day 9 (Figure 4A) (*P*<0.01). This indicates Sox9 inhibits BMP2-induced early osteogenic differentiation.

Moreover, we analyzed the effect of Sox9 on late osteogenic markers OPN using immunocytochemical staining, and found that Sox9 inhibited BMP2-induced OPN expression on day 11 after transduction (Figure 4Ba). We also demonstrated that Sox9 inhibited BMP2-induced matrix mineralization using Alizarin Red staining on day 14 after transduction (Figure 4Bb). These indicate that Sox9 inhibits BMP2-induced late osteogenic differentiation of MSCs.

**Figure 5:**
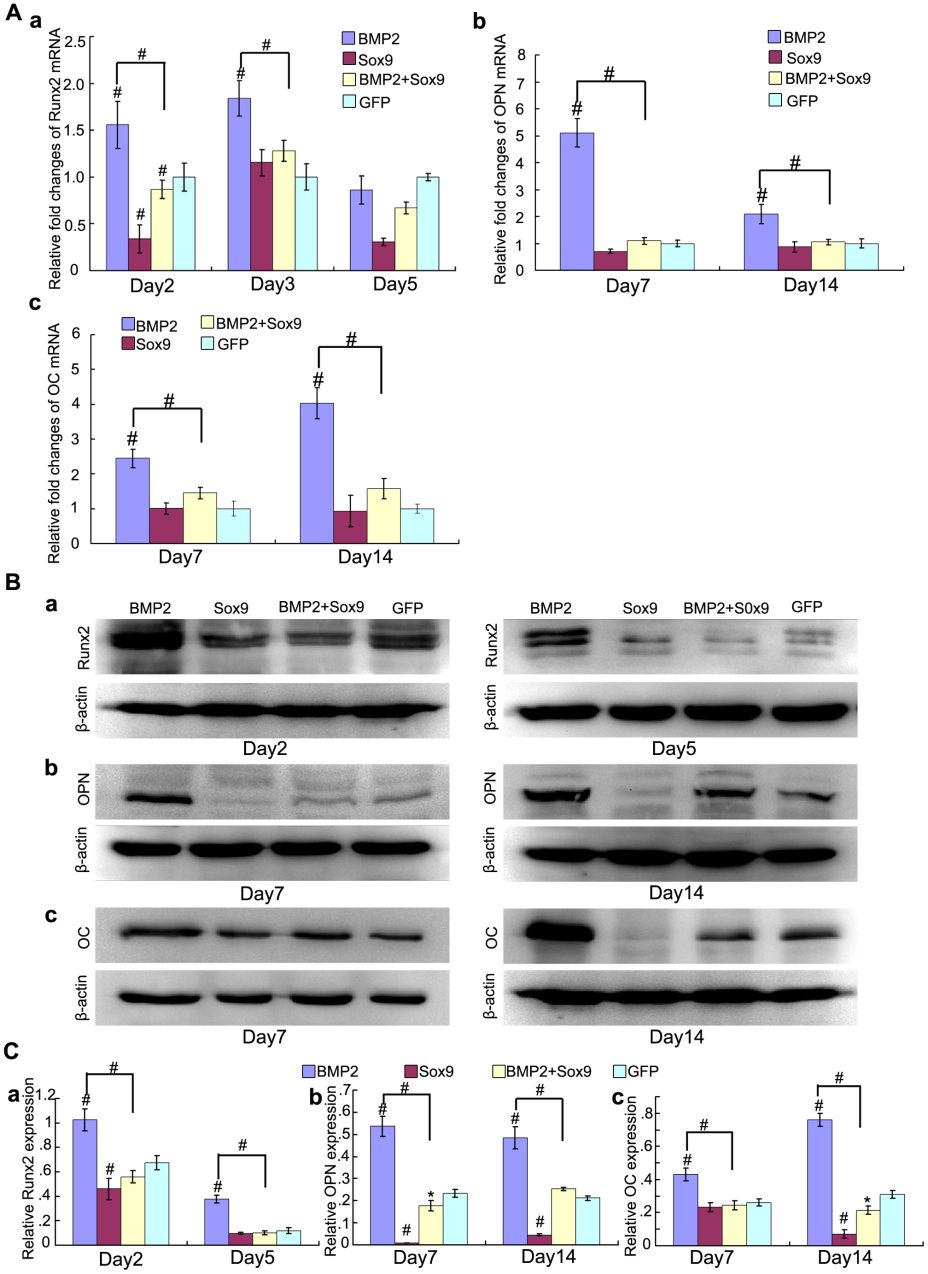
Sox9 inhibits BMP2-induced osteogenic markers expression in MSCs in vitro. (A): Real-time PCR for the expression of early osteogenic differentiation gene Runx2 (Aa), late osteogenic gene osteocalcin (OC) and osteopontin (OPN) (b, c) were conducted at indicated time points after infection with AdGFP, AdBMP2, and/or AdSox9, using GAPDH as a house keeping gene. (B): Western blot for the expression of Runx2 (a), OPN (b) and OC (c) were conducted at indicated time points after transduction of indicated recombinant adenoviruses, respectively. (C) Relative protein expression was analyzed by quantity one software using β-actin as controls respectively. The results are expressed as mean±SD of triplicate experiments, *P<0.05, ^#^ *P<0.01*.

We further determined the effect of Sox9 on BMP2-induced osteogenic gene and protein expression. Runx2 is the key transcription factor of osteogenesis, and functions at the early stage of osteogenic differentiation. We found that Sox9 inhibited Runx2 mRNA expression at early stage (day 2 and day 3) of MSCs differentiation (Figure 5Aa), and similar results were detected on protein level (Figure 5Ba, Ca) (*P*<0.01). We also detected that Sox9 inhibited BMP2-induced late osteogenic differentiation marker OPN and OC at both mRNA and protein level on day 7 and day 14 (Figure 5A, 5B, 5C) (*P*<0.01).

These results show that Sox9 inhibits BMP2-induced early and late osteogenic differentiation of MSCs in vitro.

### Sox9 Potentiates BMP2-induced Cartilage Formation and Inhibits BMP2-induced Endochondral Ossification in MSCs Implantation in vivo

**Figure 6:**
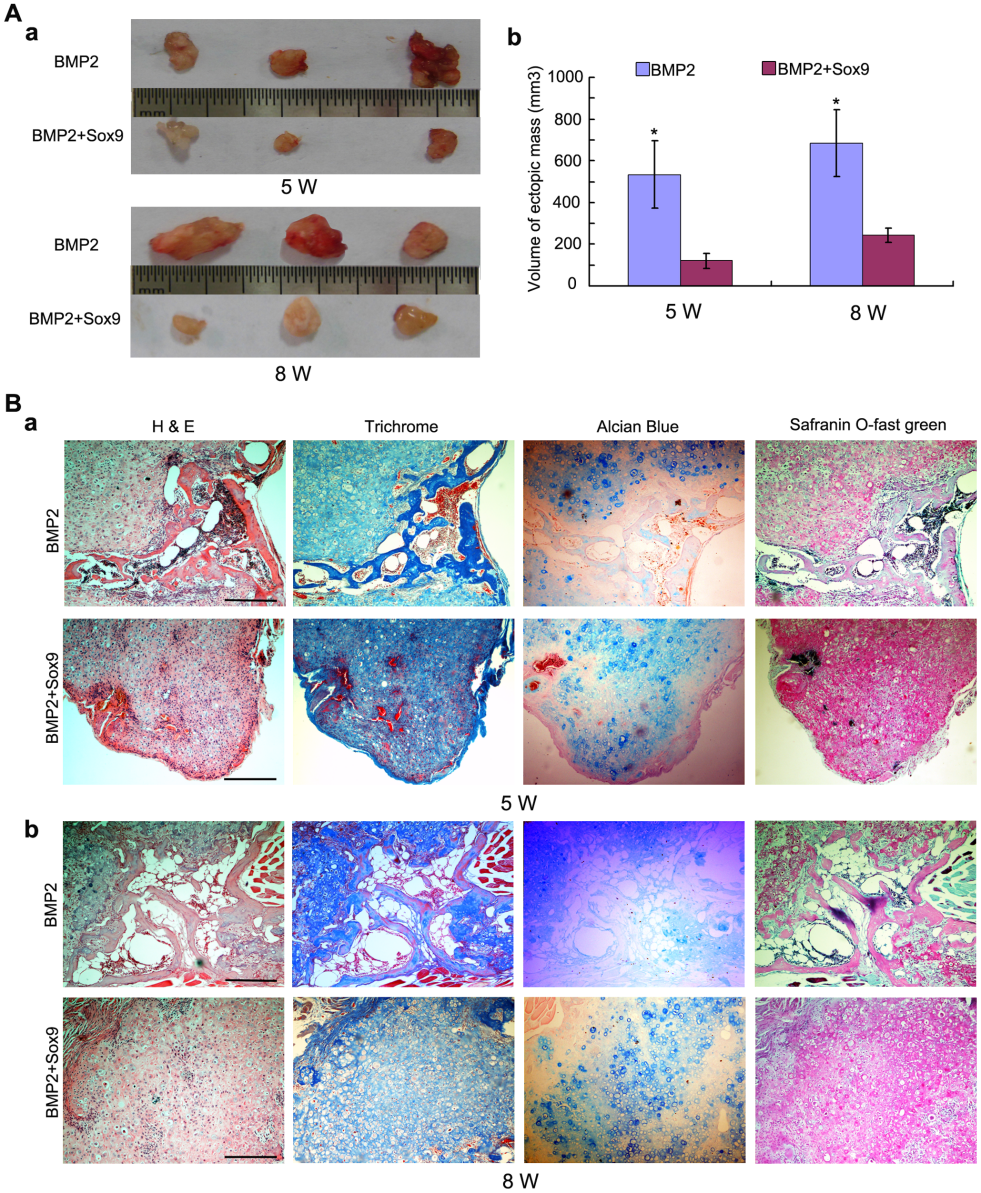
Sox9 potentiates BMP2-induced cartilage formation and inhibits BMP2-induced endochondral ossification in MSCs implantation in vivo. (A): Macrographic images of ectopic masses. BMP2 or BMP2 and Sox9 co-infected C3H10T1/2 cells were implanted subcutaneously to the flanks of nude mice. Ectopic masses were retrieved at 5 weeks and 8 weeks (a). The volume of the masses was determined using vernier calipers (b). (B): Histological analysis of the retrieved samples. The retrieved samples were fixed, decalcified, paraffin-embedded and subjected to H&E, Masson’s Trichrome, Alcian blue and safranin O-fast green staining. Representative imagines are shown, magnification, 100X, scale bar = 1 mm.

While the in vitro studies established that exogenous overexpression of Sox9 potentiates BMP2-induced chondrogenesis and inhibits BMP2-induced osteogenesis, it was imperative to demonstrate if overexpression of Sox9 play such a role in vivo. Using our previously established stem cell implantation assay [35], [37], [40], we injected C3H10T1/2 cells infected with AdGFP, AdBMP2, and/or AdSox9 at the same infection ratio subcutaneously into the flanks of athymic nude (nu/nu) mice for 5 weeks and 8 weeks, respectively. The cells transduced with AdGFP or AdSox9 alone failed to form any detectable masses (data not shown). The BMP2 and Sox9 co-infected cells formed cartilaginous/bony masses, which were noticeably smaller than those formed by cells infected by BMP2 alone (Figure 6A). On histological examination, masses formed in BMP2 transduced groups showed both bony and cartilaginous component. Masses in BMP2 transduced cell groups showed some mature bone matrices and trabeculae with the presence of a significant number of chondrocytes at week 5 (Figure 6Ba). The trabeculae turned thicker and more bone matrices formed with the presence of a significant number of hypertrophy chondrocytes at week 8 (Figure 6Bb). This indicates that BMP2 not only induce MSCs chondrogenic and osteogenic differentiation, but also stimulates endochondral ossification. On the other hand, masses formed by BMP2 and Sox9 co-infected cells showed a large number of chondrocytes with no obvious trabeculae formation both at week 5 and week 8. This indicates that Sox9 inhibits BMP2-induced osteogenesis and endochondral ossification. Masson’ trichrome, alcian blue, and safranin O-fast green staining were also used to analyze the components of the masses, which confirmed Sox9 potentiates BMP2-induced cartilage formation and inhibits BMP2-induced osteogenesis and endochondral ossification (Figure 6).

### Sox9 Promotes Expansion of the Proliferating Chondrocyte Zone and Inhibits BMP2-induced Chondrocyte Hypertrophy and Ossification in Fetal Limb Explant Cultures

**Figure 7:**
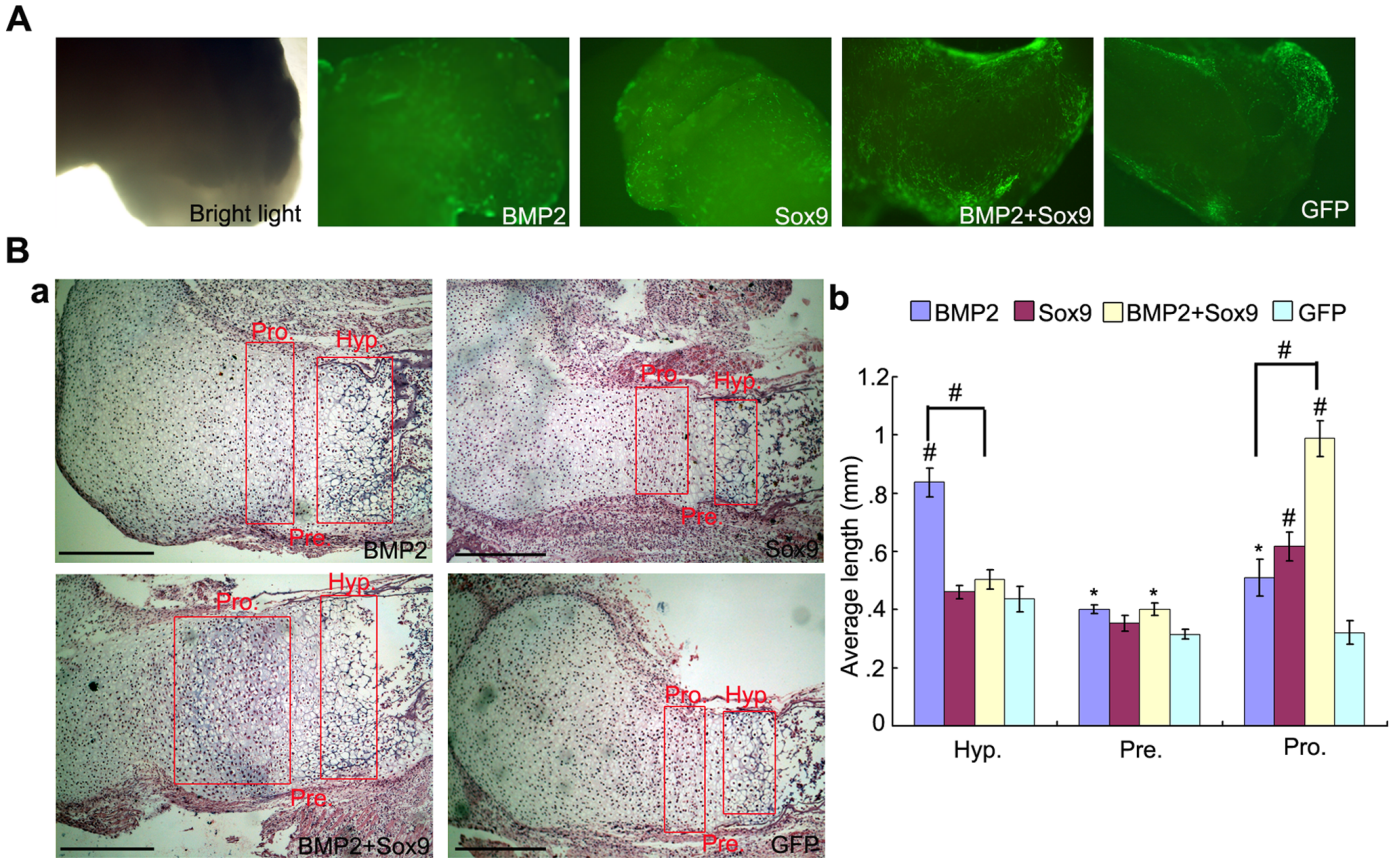
Sox9 promotes expansion of the proliferating chondrocyte zone and inhibits BMP2-induced chondrocyte hypertrophy and ossification in organ cultures. (A): Mouse E18.5 forelimbs (n = 4 each group) were harvested and transduced with AdGFP, AdBMP2, and/or AdSox9. The forelimbs were cultured in organ culture medium and the transduction efficiency was visualized under bright light and fluorescence microscope (40X). (B): Histological analysis of the cultured forelimbs. The forelimbs were fixed, decalcified, paraffin-embedded and subjected to H&E staining. Representative imagines are shown (a), magnification, 100X. The average length of the hypertrophic zones, prehypertropic zones and proliferating zones were also determined by using Image J software (b). Hyp = hypertrophic chondrocyte zone, Pre = pre-hypertrophic chondrocyte zone, Pro =  proliferating chondrocyte zone. *P<0.05, ^#^ *P<*0.01, scale bar = 1 mm.

After the in vitro and in vivo tests, we also explored the effect of Sox9 on skeletal development using the fetal limb culture assay. The skinned fetal limbs were isolated from mouse E18.5 perinatal embryos and cultured in the organ culture medium in presence of AdGFP, AdBMP2, and/or AdSox9 for 14 days. The limbs were infected with indicated recombinant adenoviruses effectively at day 5 (Figure 7A). On histological examination, both BMP2 and Sox9 induced chondrocytes proliferation and condensation. However, only BMP2 induced chondrocyte hypertrophy and ossification. When the limbs were co-infected with AdBMP2 and AdSox9, the proliferating chondrocyte zone was expanded with no obvious expansion of hypertrophic chondrocyte zone (Figure 7Ba). Quantitative analysis of the histologic data also indicated combined treatment of BMP2 and Sox9 had the largest length of proliferating chondrocyte zone (*P*<0.01), while BMP2 alone exhibited the largest length of hypertrophic chondrocyte zone (*P*<0.01) (Figure 7Bb). These results suggest that BMP2 and Sox9 act synergistically to induce chondrocytes/chondroblasts proliferation and condensation, and Sox9 inhibits BMP2-induced chondrocyte hypertrophy and ossification in fetal limb explant culture.

## Discussion

Gene-enhanced tissue engineering cartilage is a promising strategy for cartilaginous pathologies. However, it is very difficult to acquire seed cells with sufficient biological activity [41], [42]. We demonstrated here that exogenous overexpression of Sox9 significantly enhanced BMP2-induced chondrogenic differentiation and cartilage formation, while it also inhibited BMP2-induced osteogenic differentiation and endochondral ossification. Thus, overexpression of Sox9 in BMP2 induced chondrogenic differentiation may result in stable chondrogenic phenotype of MSCs.

Recombinant human bone morphogenetic protein 2 (rhBMP-2) has been approved for treating acute, open tibial shaft fractures by FDA [43]. BMP2 also exhibits high chondrogenic activity at early stage of endochondral ossification of MSCs. However, BMP2-induced osteogenic activity and endochondral ossification affect the maintenance of hyaline cartilage phenotype [44], [45]. Such limitations can be overcome by inhibiting BMP2-induced osteogenic differentiation and endochondral ossification of MSCs through directing BMP2 stimulated MSCs toward chondrogenic lineage with chondrogenic factors, such as Sox9. We found that BMP2 showed both high chondrogenic and osteogenic differentiation activity in vitro. Interestingly, overexpression of Sox9 enhanced BMP2-induced chondrogenesis and inhibited BMP2-induced osteogenesis (Figures 2–5). We further evaluate the effect of overexpression of Sox9 on BMP2-induced MSCs differentiation in vivo, the MSCs subcutaneous implantation assays showed that BMP2 induced MSCs chondrogenic differentiation, and mediated endochondral ossification in a time-dependent manner, which is in agreement with the previous studies [45], [46], [47]. When transduction with Sox9 occurred, endochondral ossification was inhibited, and hyaline cartilage was maintained (Figure 6). Finally, we explored the effect of Sox9 on skeletal development in fetal limb culture assay. Fetal limb culture could mimic the progress of chondrogenesis and endochondral ossification in vitro [37], [38], [48]. Similar with the effect of Sox9 in vivo, which showed Sox9 directs hypertrophic maturation and blocks osteoblast differentiation of growth plate chondrocyte, using a doxycycline-inducible Cre transgene and Sox9 conditional null alleles in the mouse [49], our data showed that Sox9 acted synergetically with BMP2 to expend proliferating chondrocyte zone and inhibited BMP2 induced chondrocytes hypertrophy, and ossification. Based on these results, it was concluded that Sox9 can effectively potentiate BMP2-induced chondrogenic differentiation of MSCs, as well as inhibit BMP2-induced osteogenic differentiation, and endochondral ossification of MSCs both in vitro and in vivo. Therefore, exogenous overexpression of Sox9 in BMP2-induced differentiation of MSCs may be an efficient way for cartilage tissue engineering.

Sox family was originally identified in Sry proteins, the male sex-determination transcription factor, a gene localized on the Y chromosome. It has 12 groups, and along with Sox8 and Sox10, Sox9 belongs to group E. The Sox5, Sox6, and Sox13 belong to group D [25]. Sox transcription factors act as architecture organizers. Sox5, Sox6, and Sox9, which are known as Sox trio, worked in coordination during the chondrogenesis [25], [50], [51]. Venkatesan et al. [52] reported that Sox9 gene transfer through replication-defective recombinant adeno-associated virus (rAAV) vectors can induce human MSCs chondrogenic differentiation and decrease the expression of osteogenic differentiation markers for 21 days. Cucchiarini et al. [53] also showed a process of cartilage defect repair in rabbits’ knee joints using rAAV as a gene transfer tool. Cao, L et al. [54] showed that implantation of Sox9 modifying MSCs in a polyglycolic acid (PGA) scaffold resulted in better repair of knee osteochondral defect in rabbit using recombinant adenovirus mediated gene transfer. However, it is also reported that Sox9 alone was insufficient to induce MSCs chondrogenic differentiation, but required other growth factors, such as Sox5, Sox6, IGF1, FGF or TGF-β [20], [50], [55], [56]. Our study indicated that transient overexpression of Sox9 using adenovirus vector was insufficient to induce chondrogenic differentiation of MSCs. However, overexpression of Sox9 could potentiate BMP2-induced chondrogenesis and cartilage formation.

Runx2 is a transcription factor essential for BMP2-induced bone formation, endochondral ossification, and vascular invasion [10], [23], [24], [31], [57]. However, Sox9 repress Runx2 expression by promoting transcriptional repressor Bapx1 expression [30]. In agreement with the other previous reports regarding the effect of Sox9 on Runx2 expression, we found that exogenous overexpression of Sox9 in BMP2-induced osteogenic differentiation of MSCs showed a significant decrease in the levels of Runx2 expression, sequentially with delayed osteogenic differentiation, and endochondral ossification. Apart from overexpression of Sox9, silencing or removing Runx2 might achieve a similar outcome in BMP2-induced MSCs differentiation. Kawato [58] et al found that Nkx3.2-induced suppression of Runx2 is crucial for the maintenance of chondrocyte phenotypes. Lin, L [59] et al showed that endochondral bone formation would be inhibited by silencing Runx2 in trauma-induced heterotopic ossification. Yoshida [60] et al showed that chondrocyte differentiation was inhibited depending on the dosages of Runx2, and Runx2 (−/−) mice showed a complete absence of chondrocyte maturation. Also noteworthy, it is reported that Runx2 is essential for chondrogenesis. Kim et al [61] found that Runx2 plays an important role in Ihh signaling, which induces early chondrogenesis consist of mesenchymal cell condensation, proliferation and differentiation into chondrocytes at the early stage of embryogenesis. Therefore, it may be a more efficient way to suppress the expression of Runx2 rather than completely removing it for cartilage tissue engineering.

Articular cartilage is an avascular, aneural tissue and lacks lymphatic drainage, which is composed of chondrocytes and cartilage matrix. It is essential for cartilage tissue engineering to retain the hyaline cartilage phenotype. However, chondrogenesis and endochondral ossification are tightly coupled and well coordinated during bone and cartilage formation [62]. The activation of endochondral ossification results in failure of maintaining the hyaline cartilage phenotype. The TGF-β, BMPs, and FGFs have been reported for their ability to direct MSCs towards the chondrocyte lineage. Yet these growth factors led to undesirable endochondral ossification or ectopic ossification [13], [14], [16], [43], [63], [64]. Sox9 as a master transcription factor for chondrogenesis also delayed BMP2 induced bone formation of MSCs, and endochondral ossification through repress Runx2 expression, and thus plays a crucial role in cartilage formation and cartilaginous pathologies healing [49], [53]. We confirmed that Sox9 mediated inhibition of osteogenic differentiation plays an important role in BMP2-induced cartilage formation and keeping hyaline cartilage phenotype.

Although it is well known that chondrogenesis and endochondral ossification are tightly coupled in cartilage and bone formation, it is unclear how these processes are linked in BMP2 induced MSCs differentiation. Our study demonstrated that exogenous overexpression of Sox9 potentiates BMP2-induced MSCs chondrogenesis and cartilage formation, as well as inhibits BMP2-induced MSCs osteogenesis and endochondral ossification. Thus, exogenous overexpression of Sox9 in BMP2-induced MSCs differentiation can be considered as a new strategy for cartilage tissue engineering.
