# Expression of Concern: Sox9 Potentiates BMP2-Induced Chondrogenic Differentiation and Inhibits BMP2-Induced Osteogenic Differentiation

**DOI:** 10.1371/journal.pone.0249684

**Published:** 2021-04-01

**Authors:** The PLOS ONE Editors

Following the publication of this article [1], concerns were raised regarding panels presented in Figs 1, 2 and 5. Specifically,

- In the left panel of Fig 1C, the left side of the bands do not appear to align correctly with the right sides of the bands.
- The Day 5 anti-β-actin panel of Fig 1Db appears similar to the Day 5 β-actin panel of Fig 5B.
- The Sox9 panel and the GFP panel of Fig 2C partially overlap.
- The Day 2 β-actin panel of Fig 5B appears similar to the Day 14 β-actin panel of Fig 5C when flipped.

**Fig 2:**
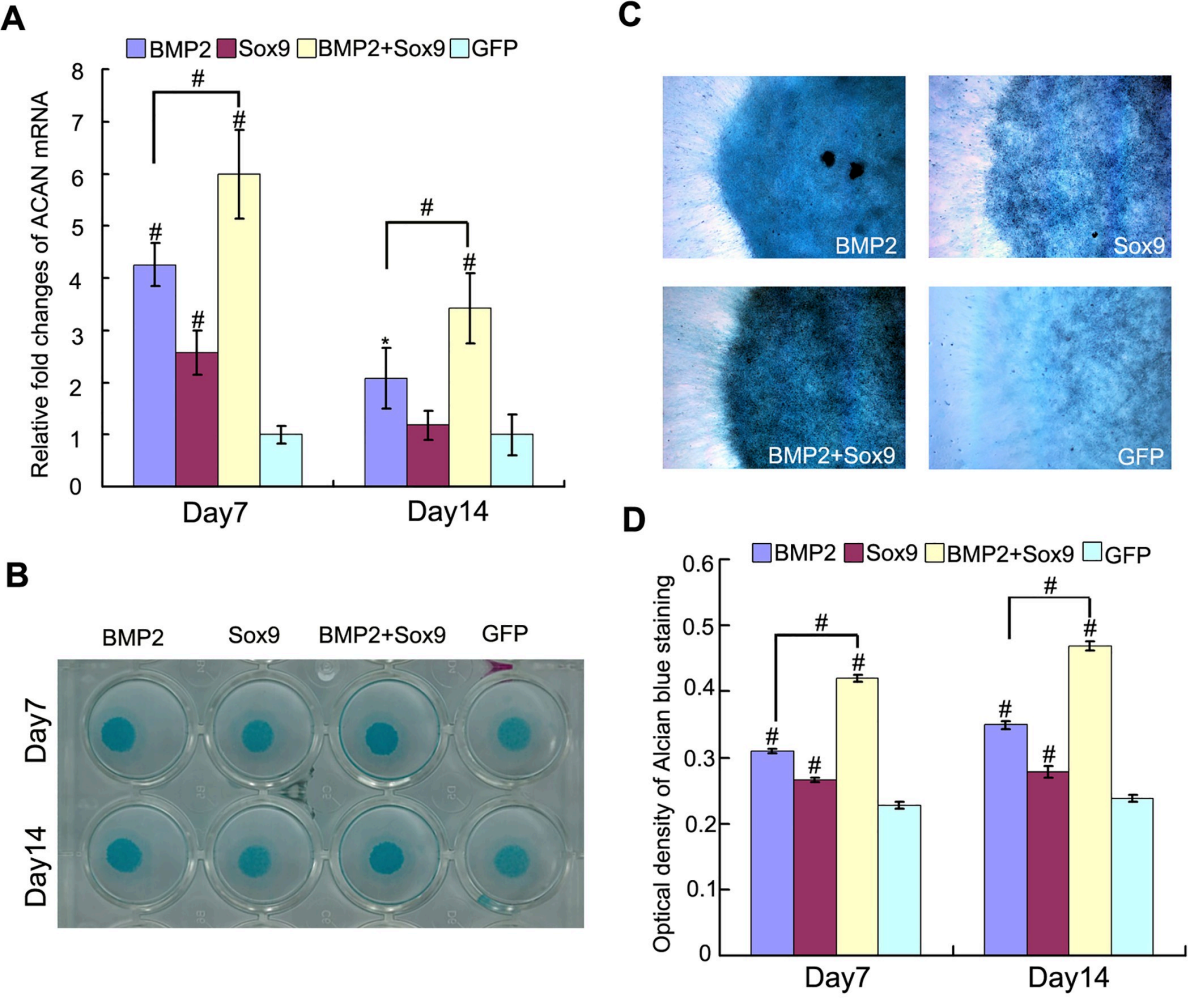
Sox9 potentiates BMP2-induced glycosaminoglycans synthesis in MSCs in micromass cultures. (A): Real-time PCR for the expression of chondrogenic differentiation marker gene ACAN were conducted on day 7 and day 14 after infection of AdGFP, AdBMP2, and/or AdSox9, using GAPDH as a house keeping gene. (B–C): Alcian blue staining for sulfated glycosaminoglycans in micromass cultures of C3H10T1/2 cells on day 7 and day 14 after transduction of indicated recombinant adenoviruses, gross observation (B) and microscope examination (C, 40X) are shown. (D): Alcian blue staining quantifying: cells were extracted with 6 M guanidine hydrochloride, Optical density of the extracted dye was measured at 630 nm. The results were expressed as mean±SD of triplicate experiments, *P<0.05, #P<0.01.

**Fig 5:**
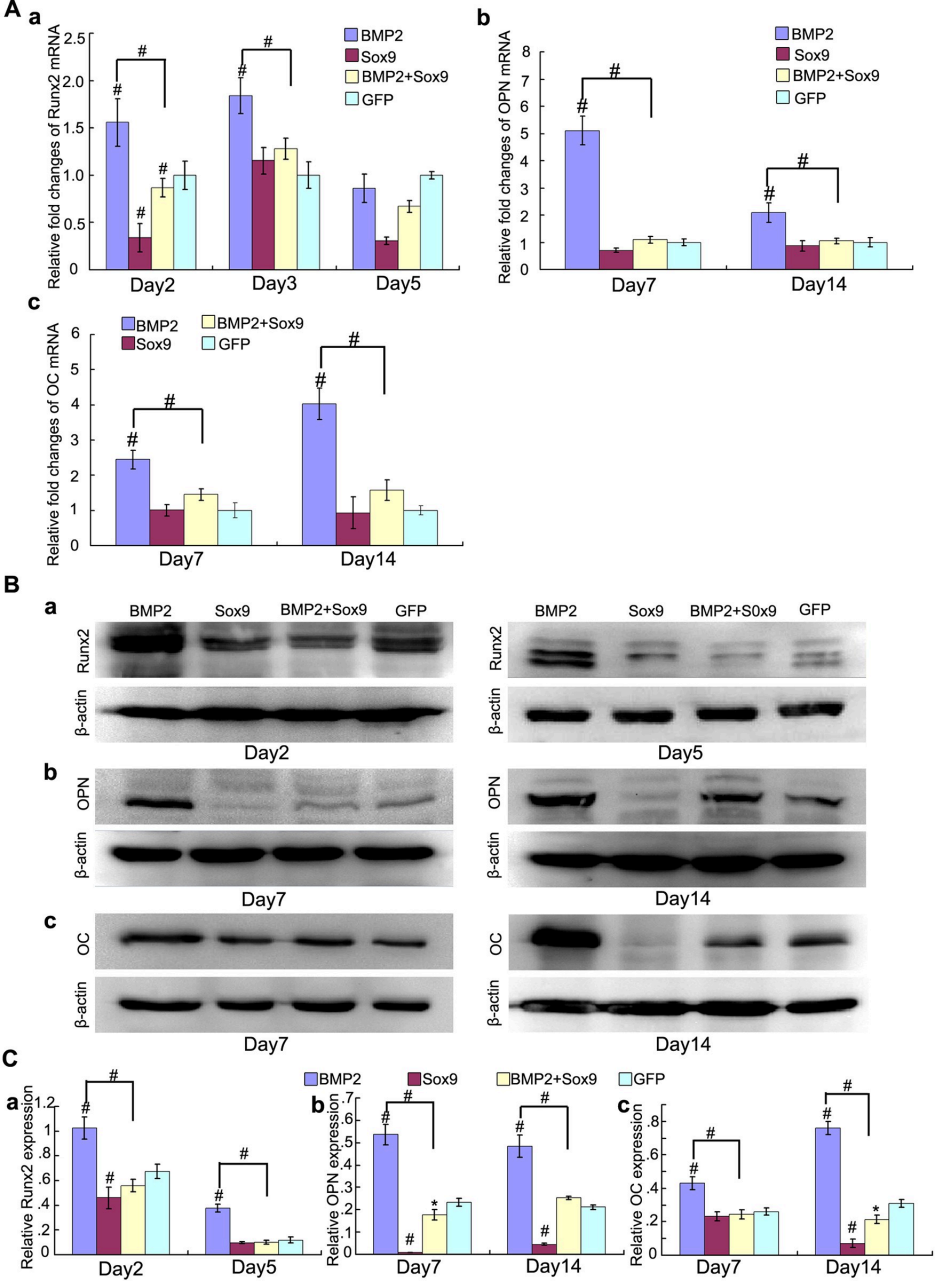
Sox9 inhibits BMP2-induced osteogenic markers expression in MSCs in vitro. (A): Real-time PCR for the expression of early osteogenic differentiation gene Runx2 (Aa), late osteogenic gene osteocalcin (OC) and osteopontin (OPN) (b, c) were conducted at indicated time points after infection with AdGFP, AdBMP2, and/or AdSox9, using GAPDH as a house keeping gene. (B): Western blot for the expression of Runx2 (a), OPN (b) and OC (c) were conducted at indicated time points after transduction of indicated recombinant adenoviruses, respectively. (C) Relative protein expression was analyzed by quantity one software using β-actin as controls respectively. The results are expressed as mean±SD of triplicate experiments, *P<0.05, #P<0.01.

The corresponding author has provided the original images underlying Fig 1C and indicated that black/white inversion was performed to improve the presentation of the results. The uncropped underlying figures provided in the S1 File below support the panel presented in Fig 1C suggesting the irregularities in this figure are compression artifacts. The uncropped underlying blots and individual level data to support the published results presented in Fig 1D are summarised in the S2 and S3 Files below.

The similarity between the Sox9 and GFP panel in Fig 2C is the result of an inadvertent error during figure assembly, whereby the wrong panel was used to represent the GFP results. The corresponding author provided an updated Fig 2 presenting the correct GFP panel. The individual data supporting the results presented in the updated Fig 2 are summarised in the S4 File below.

The similarity between the western blot panels in Fig 1D and Fig 5B are the result of inadvertent errors during figure assembly. The corresponding author has indicated that the panels used to represent the Fig 5Ba Day 5 β-actin results and the Fig 5Bc Day 14 β-actin results are incorrect and proved an updated Fig 5 presenting the correct panels. In addition, the corresponding author indicated that the Fig 5Ba Day 5 Runx2 panel was presented upside-down. The underlying images and individual level data supporting the results presented in the updated Fig 5 are summarised in the supporting information S5–S8 Files.

The PLOS ONE Editors issue this Expression of Concern to notify readers of the above concerns and relay the supporting data and updated figures provided by the corresponding author.

## Supporting information

S1 File Blots underlying Fig 1C. (TIF)

S2 File Blots underlying Fig 1D. (TIF)

S3 File Individual level data underlying Fig 1D. (XLS)

S4 File Individual level data underlying Fig 2A and 1D. (XLSX)

S5 File Blots underlying Fig 5Ba. (TIF)

S6 File Blots underlying Fig 5Bb. (TIF)

S7 File Blots underlying Fig 5Bc. (TIF)

S8 File Individual level data underlying Fig 5A and 5C. (XLSX)
