# Supplementary figures and images for: Expression of Concern: Sox9 Potentiates BMP2-Induced Chondrogenic Differentiation and Inhibits BMP2-Induced Osteogenic Differentiation
Source: PLoS One. 2021 Apr 1;16(4):e0249684. doi: 10.1371/journal.pone.0249684 (PMC8016310; doi:10.1371/journal.pone.0249684)

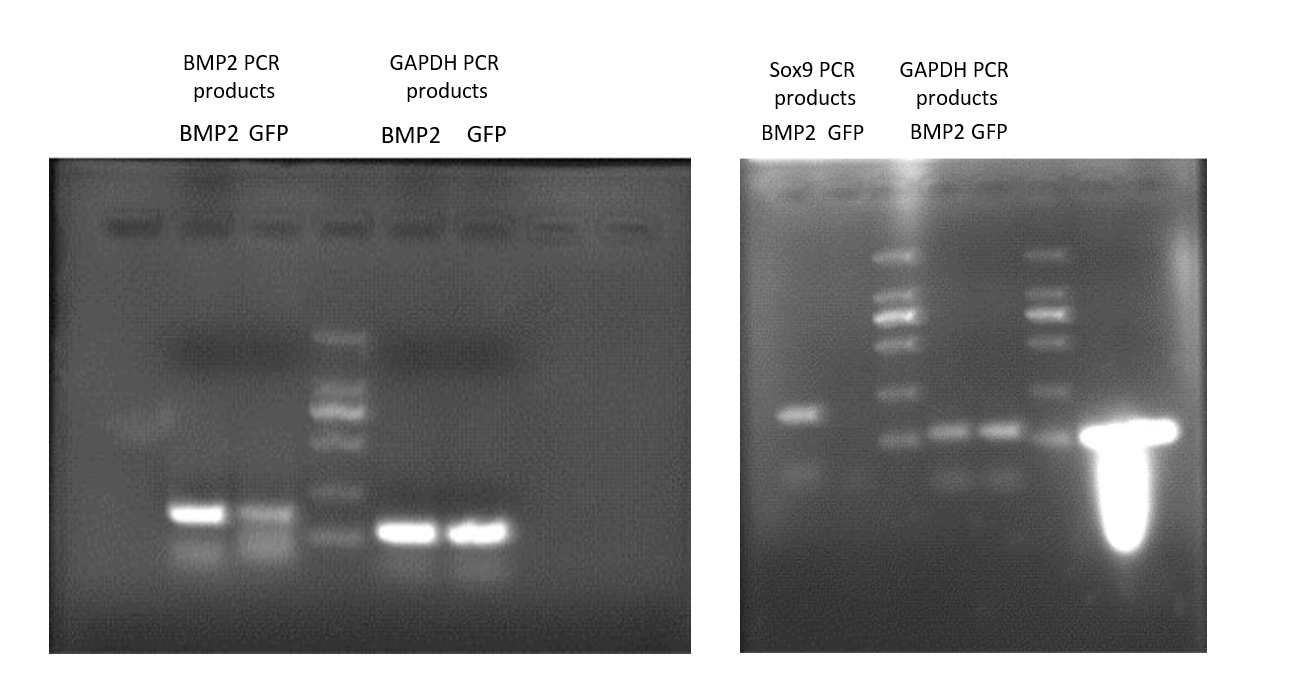

Supplement: S1 File — (TIF) [file pone.0249684.s001.tif]

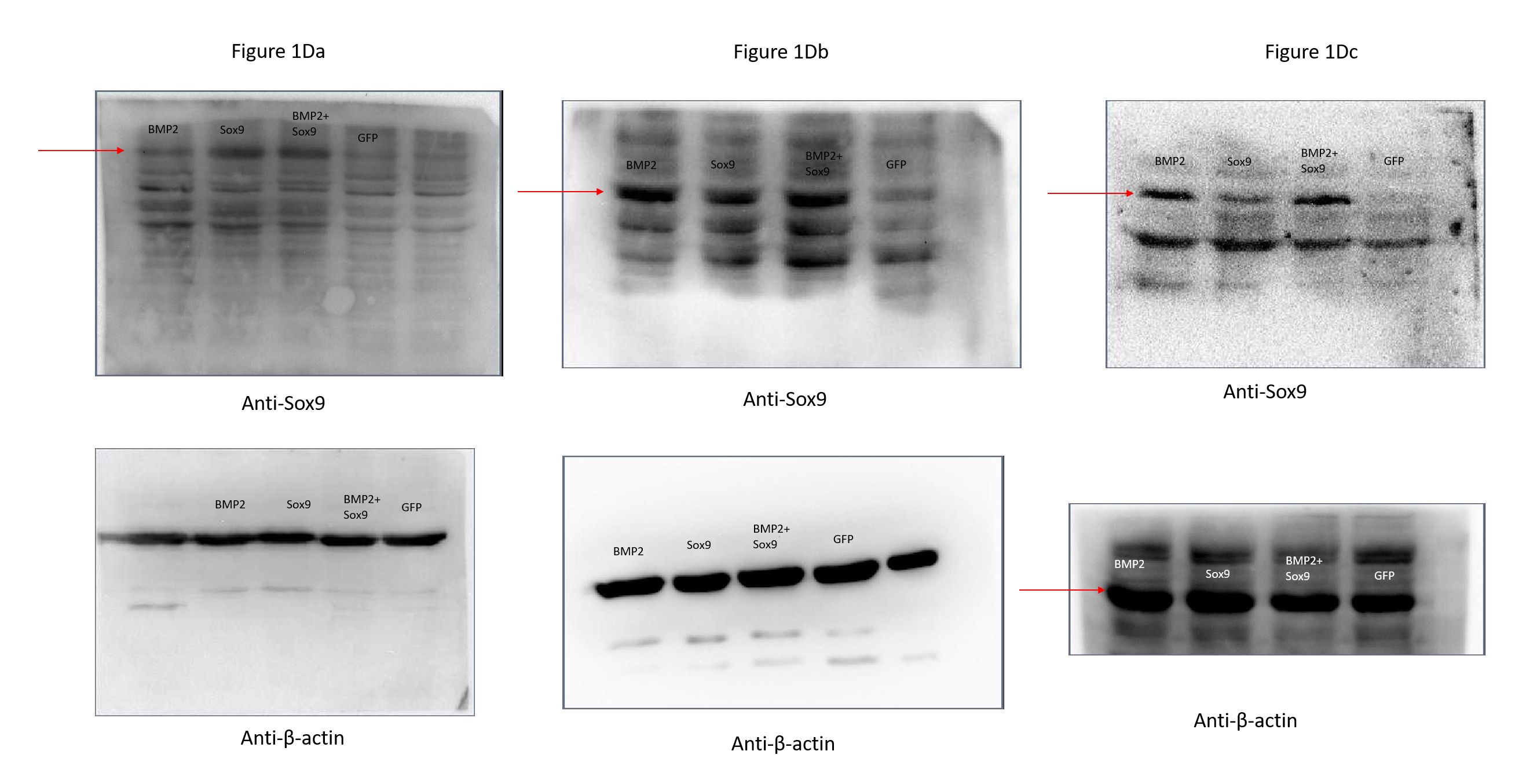

Supplement: S2 File — (TIF) [file pone.0249684.s002.tif]

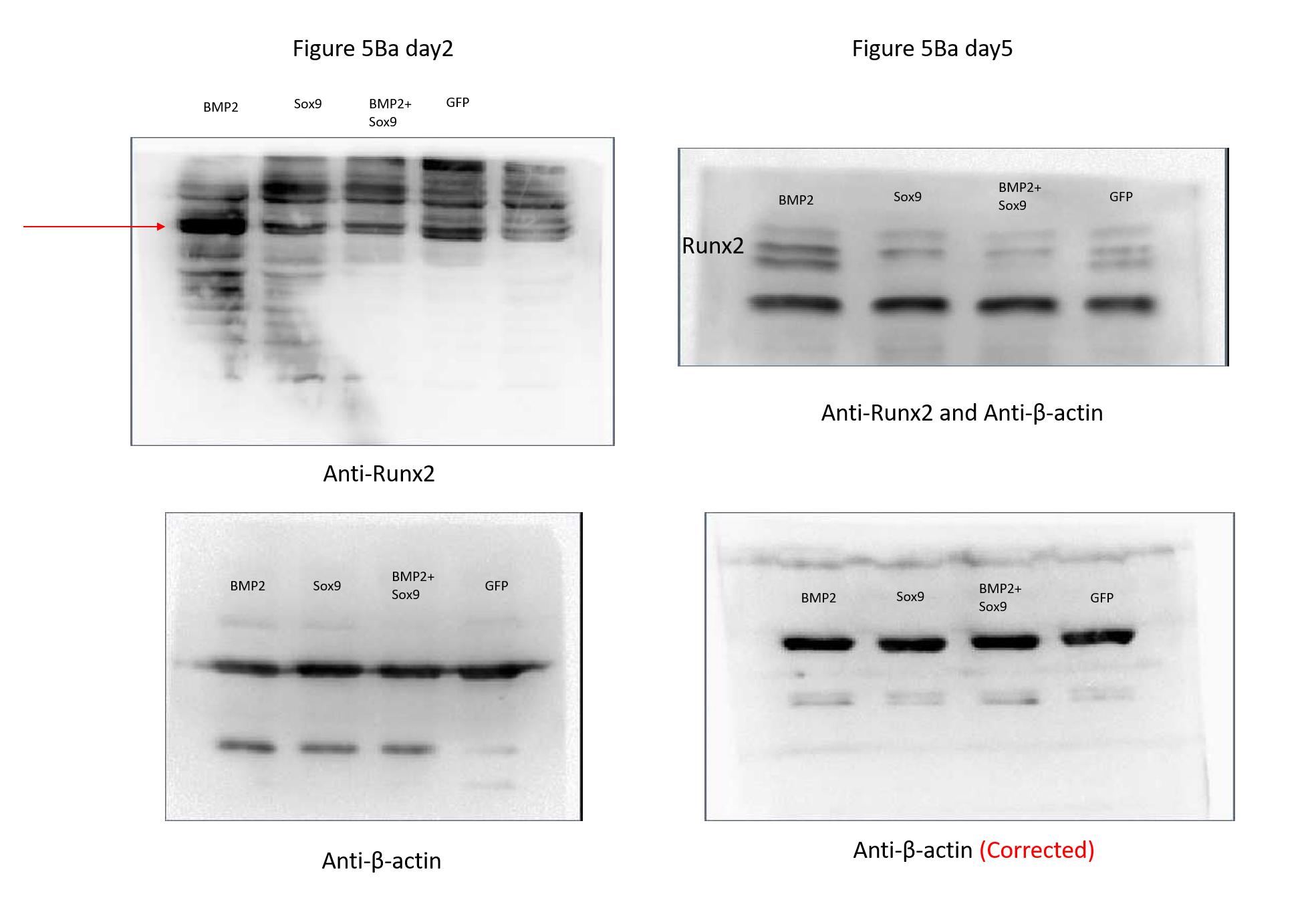

Supplement: S5 File — (TIF) [file pone.0249684.s005.tif]

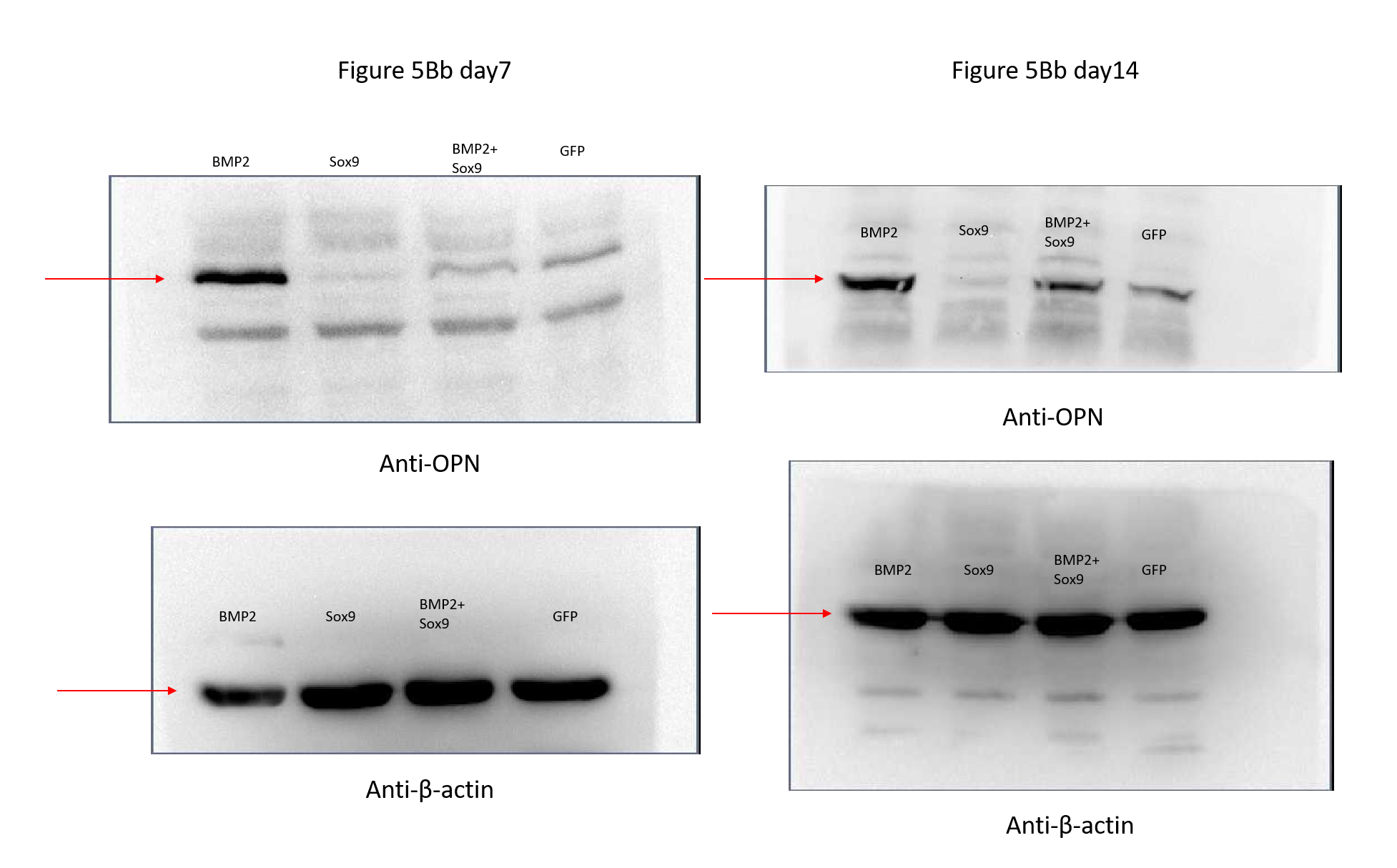

Supplement: S6 File — (TIF) [file pone.0249684.s006.tif]

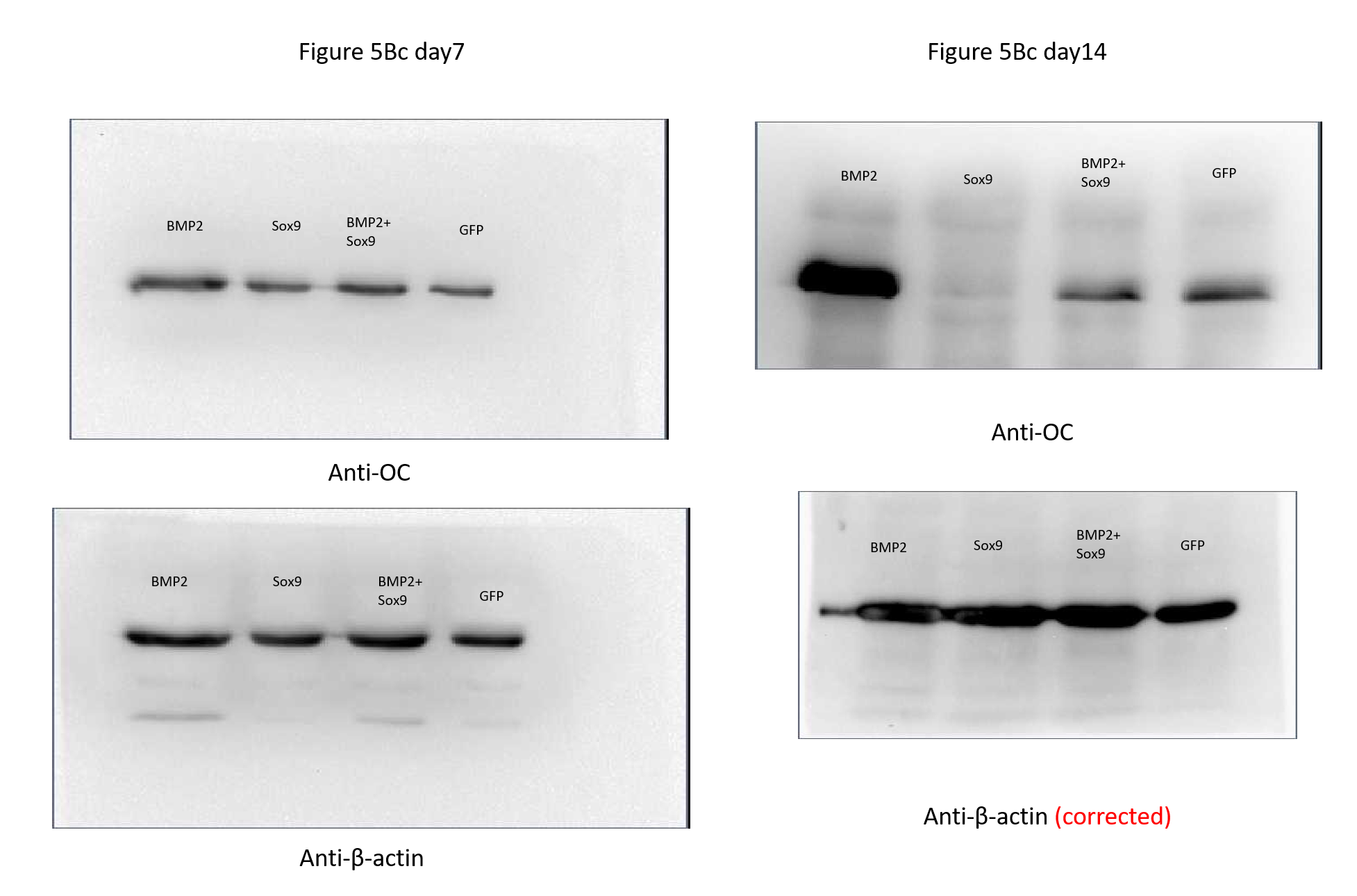

Supplement: S7 File — (TIF) [file pone.0249684.s007.tif]
